# A Genome-Wide Screening of Potential Target Genes to Enhance the Antifungal Activity of Micafungin in *Schizosaccharomyces pombe*

**DOI:** 10.1371/journal.pone.0065904

**Published:** 2013-05-30

**Authors:** Xin Zhou, Yan Ma, Yue Fang, Wugan gerile, Wurentuya Jaiseng, Yuki Yamada, Takayoshi Kuno

**Affiliations:** 1 Department of Oncology, the First Affiliated Hospital of Liaoning Medical University, Jinzhou, China; 2 Division of Molecular Pharmacology and Pharmacogenomics, Department of Biochemistry and Molecular Biology, Kobe University Graduate School of Medicine, Kobe, Japan; 3 Department of Pharmacology, School of Pharmaceutical Sciences, China Medical University, Shenyang, China; University of Aberdeen, United Kingdom

## Abstract

Micafungin is a non-reversible inhibitor of 1, 3-*β*-D-glucan synthase and interferes with fungal cell wall synthesis. Clinically, micafungin has been shown to be efficacious for the treatment of invasive *candidiasis* and invasive *aspergillosis*. However, considering its relatively restricted antifungal spectrum, combination therapy with micafungin plus other agents should be considered in critically ill patients. To identify potential therapeutic targets for syncretic drug combinations that potentiate micafungin action, we carried out a genome-wide screen for altered sensitivity to micafungin by using the model yeast *Schizosaccharomyces pombe* mutant library. We confirmed that 159 deletion strains in the library are micafungin sensitive and classified them into various functional categories, including cell wall biosynthesis, gene expression and chromatin remodeling, membrane trafficking, signaling transduction, ubiquitination, ergosterol biosynthetic process and a variety of other known functions or still unknown functions. On the other hand, we also investigated the growth inhibitory activities of some well-known drugs in combination with micafungin including antifungal drug amphotericin B, fluconazole and immunosuppressive drug FK506. We found that amphotericin B in combination with micafungin showed a more potent inhibitory activity against wild-type cells than that of micafungin alone, whereas fluconazole in combination with micafungin did not. Also, the immunosuppressive drug FK506 showed synergistic inhibitory effect with micafungin on the growth of wild-type cells, whereas it decreased the inhibitory effect of micafungin in Δ*pmk1* cells, a deletion mutant of the cell wall integrity mitogen-activated protein kinase (MAPK) Pmk1. Altogether, our findings provide useful information for new potential drug combinations in the treatment of fungal infections.

## Introduction

Invasive fungal infections are important causes of morbidity and mortality in immunocompromised patients, particularly high-risk populations, such as those receiving cancer chemotherapy and hematopoietic stem cell transplantation [1], [2]. *Candida* and *Aspergillus* species are the most common causes of invasive fungal infections, accounting for 70–90% and 10–20% of all invasive mycoses, respectively [3]. Micafungin, an inhibitor of the enzyme 1, 3-*β*-D-glucan synthase, was approved as a promising echinocandin against *Candida* and *Aspergillus* species by the US Food and Drug Administration [4]. However, given the restricted antifungal spectrum of micafungin [5], clinicians have shown great interest for using combinations of micafungin and other antifungal agents in the treatment of invasive fungal infections.

The model yeast *Schizosaccharomyces pombe* (*S. pombe*) is a single-celled living archiascomycete fungus that shares many features with pathogenic fungi. According to the results of alkali treatment and methylation analysis, there is 46–54% 1, 3-*β*-D-glucan in the cell wall of *S. pombe* [6], which make it an excellent model system to study the mechanisms that influence the antifungal activity of micafungin. On another hand, we have performed a genome-wide screen in *S. pombe* for altered sensitivity to antifungal drugs, including clotrimazole and terbinafine that target ergosterol biosynthesis [7]. In this study we aimed to identify genes affecting sensitivity to micafungin.

The mode of actions of antifungal agents are based on the inhibition of molecular targets involved in some biological processes including ergosterol biosynthesis for azole derivatives, cell membrane permeability for polyenes, and cell wall integrity for echinocandins [8], [9]. To identify potential therapeutic targets for agents that would increase the antifungal effect of micafungin, we performed a genome-wide screen using *S. pombe* haploid deletion library to search for the mutants that display hypersensitivity to micafungin. Our results showed that genes involved in complex biological processes contribute to increase the antifungal activity of micafungin, which provides useful information for further research of the synergistic enhancers of micafungin in clinical practice.

Furthermore, we investigated the growth inhibitory activities of some well-known drugs in combination with micafungin. We found that the polyene antifungal drug amphotericin B (AmB) effectively increased the growth inhibitory activity of micafungin against wild-type cells, whereas the inhibitors of ergosterol biosynthesis including azoles and terbinafine did not. Notably, immunosuppressive drug FK506 (tacrolimus) exhibited synergistic activity with micafungin against wild-type cells, however, contrary to our assumption, FK506 decreases the inhibitory activity of micafungin against Δ*pmk1* cells, a deletion mutant of the cell wall integrity MAPK Pmk1, in a calcineurin-dependent manner.

## Materials and Methods

### Deletion library construction, media, genetic and molecular biology methods

Heterozygous diploid deletion strains were constructed and supplied by BiONEER (South Korea) using the method of PCR-based targeted gene deletion with a genetic background of *h^+^ leu1-32 ura4-D18 ade6-M210* or *-M216* [10]. The haploid deletion library used in this study consists of 3004 mutants representing approximately 71.8% of the non-essential *S. pombe* genes. The other strains used in this study are listed in Table S2.

Standard media, notation and genetic methods have been described previously [11]. YES (rich yeast extract with supplements) plates are supplemented with 225 mg/l adenine, histidine, leucine, uracil, and lysine.

### Deletion library screens for micafungin sensitivity

The deletion library was provided on agar plates and stamped in a 96-well format. Prior to performing the experiment, the library was transferred to YES plates at 27°C. The log-phase cells were streaked onto YES plates with or without 0.5 µg/ml micafungin (Astellas Pharma Inc. Japan) and incubated at 27°C for 4 days for preliminary screen. Deletion mutants that exhibited growth inhibition in the preliminary screen were selected to carry out the secondary and tertiary screens using a representative dilution-series spot assay. The wild-type cells and selected mutants were grown to saturation in liquid medium YES at 27°C. The cultures were then resuspended in fresh YES medium to give an optical density (OD) at 660 nm of 0.3, corresponding to about 10[7] cells/ml, and serially diluted to concentrations of 1×10^−1^ to 1×10^−4^. The 5 µl samples of 10-fold serial dilutions of each yeast cell culture were spotted onto YES plates with or without 0.5 µg/ml micafungin, and incubated at 27°C for 4 days. Dilutions of micafungin-sensitive mutants were also spotted onto YES plates containing 20 µg/ml AmB (Bristol Myers Squibb. K.K., Tokyo, Japan) and incubated at 27°C for 4 days. The growth inhibition of each mutant was scored as severe (+++), moderate (++), or mild (+).

### Cell wall digestion assay

Cell wall digestion by *β*-glucanase (Zymolyase, Seikagakukogyo, Tokyo, Japan) was performed as described previously [12]. Briefly, exponentially growing cells at 27°C were suspended at a concentration of 10[7] cells/ml. Then, cells were treated with *β*-glucanase at a concentration of 100 µg/ml at 27°C. Cell lysis was monitored by measuring OD at 660 nm.

### Bioinformatics

Database searches were performed using the National Center for Biotechnology Information BLAST network service (www.ncbi.nlm.nih.gov) and the Sanger Center *S. pombe* database search service (www.sanger.ac.uk).

## Results and Discussion

### Identification of genes affecting the sensitivity to micafungin

**Figure 1:**
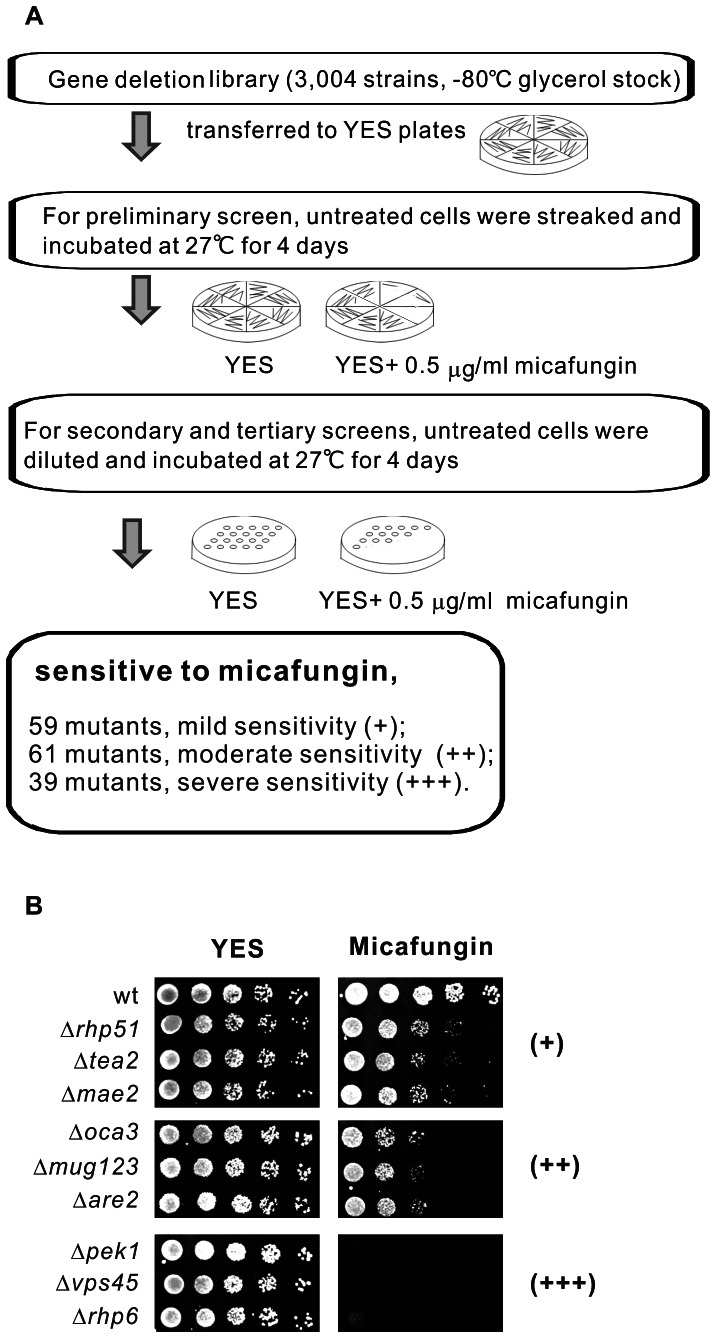
Genome-wide screening of micafungin-sensitive mutants. (A) Summary of micafungin-sensitive mutants screening. (B) Representative examples of isolated mutants that showed varying levels of sensitivity to micafungin. Wild-type cells and deletion mutants grown at log phase were spotted onto YES plates with or without 0.5 µg/ml micafungin and then incubated at 27°C for 4 days.

To determine the optimal concentration for screening, wild-type and Δ*pmk1* cells which showed micafungin hypersensitivity in our previous study [13], were streaked onto YES plates with various concentrations of micafungin. The results showed that the growth of Δ*pmk1* cells was severely impaired on YES plates containing 0.5 µg/ml micafungin where wild-type cells showed normal growth rates (data not shown). In the preliminary screen, 3004 deletion strains were streaked on YES plates containing 0.5 µg/ml micafungin. The mutants with growth inhibition were liberally selected, ensuring no sensitive mutants were missed. All these selected sensitive mutants were retested by a representative dilution-series spot assay as described in Materials and Methods and the severity of growth inhibition by micafungin was scored according to the number of spots that grew on the micafungin-containing plates: severe sensitivity (+++) indicates that no spot or the first spot could grow slowly (Figure 1B, lower panel, and Table S1), moderate sensitivity (++) indicates that the third spot could grow slowly (Figure 1B, middle panel, and Table S1), and mild sensitivity (+) indicates that the fourth spot could grow slowly (Figure 1B, upper panel, and Table S1). Among the 175 mutants that were identified to show varying levels of sensitivity to micafungin (Figure 1A and Table S1), 16 mutants that showed clear growth defect compared with wild-type cells on YES plates were excluded. Ultimately, 39 mutants were scored as severe sensitivity (+++), 61 mutants were scored as moderate sensitivity (++) and 59 mutants were scored as mild sensitivity (+) (Figure 1B and Table S1). The present screen is reliable by the same growth inhibition on micafungin containing plates of some known micafungin-sensitive mutants such as Δ*pek1* and Δ*vps45* (Figure 1B) as previously reported [13], [14]. All of these micafungin-sensitive mutants were classified by their biological functions as follows: cell wall biosynthesis, gene expression and chromatin remodeling, membrane trafficking, signaling transduction, ubiquitination, ergosterol biosynthetic process, variety of other known functions and unknown functions. Of each gene listed in Table S1, the systematic name, common gene name (if applicable), along with a brief description of the function of each gene product were also indicated. For convenience, we named the genes after their *S. cerevisiae* counterparts when the common name is not applicable. The hypersensitivity to micafungin of these mutants suggested that the antifungal effect of micafungin could be increased by inhibiting the causative genes identified.

### Genes involved in cell wall biosynthesis

The first group of gene identified corresponds to genes involved in cell wall biosynthesis. As shown in Table S1, the deletion of most of genes involved in cell wall integrity MAPK pathway exhibited severe sensitivity to micafungin, including *pmk1* ^+^, *pek1* ^+^, *rho2* ^+^, *pck1* ^+^, *rgf1* ^+^, *rga8* ^+^, and *trp1322* ^+^. They are in good agreement with previous findings from our laboratory and others [15]–[19]. It should be noted that the *rgf1* ^+^ and *rga8* ^+^ encode guanine nucleotide exchange factor (GEF) and GTPase activating protein (GAP), respectively, for the *S. pombe* Rho1 and Rho1 regulates the synthesis of 1, 3-*β*-D-glucan by activation of the 1, 3-*β*-D-glucan synthase [16]. Similar to Δ*rho2*, deletion of *rga7* ^+^, one of Rho2 GAP, also exhibited moderate sensitivity to micafungin. These results indicate that the GTP/GDP ratio of Rho GTPase is important for the regulation of the cell wall integrity and the alteration in the GTP/GDP balance of Rho might lead to micafungin sensitivity in *S. pombe* cells. Deletion of some genes involved in cell wall biogenesis also exhibited hypersensitivity to micafungin, such as the *pvg2* ^+^ and *pvg5* ^+^ genes, which are involved in the pyruvylated galactose (PvGal) biosynthetic pathway. And the *mde10* ^+^ gene, whose product Mde10 was reported important in the development of the spore envelope. Our results suggested that Mde10 might exert its effects in spores by influencing the synthesis of 1, 3-*β*-D-glucan.

### Genes involved in gene expression and chromatin remodeling

The largest group of genes identified comprises pathways involved in gene expression and chromatin remodeling. As shown in Table S1, deletion of 9 genes were identified to display severe micafungin sensitivity, including *rhp54* ^+^, *rhp6* ^+^, *ccr4* ^+^, *caf1* ^+^, *exo2* ^+^, *png1* ^+^, *pms1* ^+^, *tyw1* ^+^, and *pat1* ^+^ genes. The *rhp54* ^+^ and *rhp6* ^+^ genes are homologue of *S*. *cerevisiae RAD54* and *RAD6*, respectively. The *rhp51* ^+^ gene, deletion of which showed only mild sensitivity to micafungin, is *RAD51* homologue. These genes are proved to be involved in genetic recombination and double-strand break repair [20], [21]. The *ccr4* ^+^ and *caf1* ^+^ genes encode subunits of Ccr4-Not complex, which is thought to reduce the poly(A) tail to a short oligo(A) tract before the body of the mRNA is degraded by subsequent enzymatic activities [22]. Furthermore, in *S*. *cerevisiae*, Ccr4-Not transcriptional complex plays a positive role in *RAD51* expression [23]. Png1, a fission yeast ING (inhibitor of growth) homolog, functions upstream of DNA recombination protein Rad52 in the DNA damage response pathway and is involved in the repair of double-strand breaks in DNA during vegetative growth and meiosis [24]. Pms1 mismatch repair protein affected the pattern of microhomology-mediated end joining (MMEJ) repair. Recently, it has been demonstrated that in *S*. *cerevisiae*, deletion of the elements of SWI/SNF chromatin-remodeling complex renders cells hypersensitive to cell wall stress [25]. Here, our results showed in *S. pombe* the defects in gene expression and chromatin remodeling also affect cell wall integrity. Probably deletion of these strand exchange protein-coding genes led to defective expression of some important genes involved in cell wall integrity.

### Genes involved in membrane trafficking

Another major group of genes encode proteins involved in intracellular transport, including *vps1302* ^+^, *vps45* ^+^, *tlg2* ^+^, *ent3* ^+^, *emc1* ^+^, *imt3* ^+^, *pal1* ^+^, and *end4* ^+^. Deletion of these genes showed severe micafungin sensitivity. The *vps1302* ^+^ gene is highly conserved, with orthologs in all eukaryotic genomes that have been sequenced. In *S. cerevisiae*, *VPS13*, homologue of *vps1302* ^+^, is involved in the delivery of proteins to the vacuole [26]. The *vps45* ^+^ gene encodes Vps45, which regulates endosomal trafficking in fission yeast, binds the conserved N-terminal peptide of the syntaxin Tlg2 [27]. The *ent3* ^+^ gene is the homologue of *S. cerevisiae ENT3*, which encodes an epsin-like TGN/endosome adaptors and is involved in retrograde transport from early endosomes to the TGN (*trans*-Golgi network) [28]. The *imt3* ^+^ gene encodes one subunit of mannosyltransferase complex, which is involved in the synthesis of mannosylinositol phosphorylceramide (MIPC). The MIPC-deficient mutant exhibited pleiotropic phenotypes, including defects in cellular and vacuolar morphology, and in localization of ergosterols [29]. To our surprise, deletion of the *imt2* ^+^ gene, another mannosyltransferase encoding gene, displayed hypersensitivity to none of four antifungal drugs including micafungin, AmB, terbinafine, and clotorimazole (data not shown), indicating that Imt3 may play a more important role than Imt2 in MIPC synthesis. Pal1 and End4 are both important for maintenance of cylindrical cellular morphology. Pal1 is a membrane-associated protein, and End4 is important for efficient localization of Pal1 and appears to function upstream of Pal1 [30]. Altogether, we infer that deletion of these genes associated with membrane trafficking probably led to some cell-wall-integrity-related-proteins failing to localize to the cell surface and the medial regions.

### Genes involved in other cellular processes

Genes modulating other biological processes, such as signaling transduction, ubiquitination, and ergosterol biosynthetic process, also contribute to hypersensitivity to micafungin, upon gene deletion (Table S1). The *rrd1* ^+^ and *rrd2* ^+^ genes, are homologue of *RRD1* and *RRD2*, respectively. They encode the activators of the phosphotyrosyl phosphatase activity of protein phosphatase 2A and are involved in various signal transduction pathway including HOG1 osmotic stress response pathway [31], [32]. In our study, both Δ*rrd1* and Δ*rrd2* showed severe sensitivity to micafungin (Table S1). To our surprise, both of these two mutants also showed osmo-remedial phenotype (Table 1), which is contrary to the reported results in *S. cerevisiae* that double deletion of *RRD1* and *RRD2* causes impaired growth on sorbitol-containing medium [32], suggesting that Rrd proteins probably play some different roles in *S. pombe*. Ckbl is evolutionary conserved from yeast to humans and plays a role in mediating the interaction of casein kinase II with downstream targets and/or with additional regulators [33]. Our results suggested that casein kinase II exerts influence on establishment of cell shape by regulating protein substrates or processes associated with cell wall integrity. In addition, there are a number of mutants that also exhibited hypersensitivity to micafungin although their functions are not clear, including *svf1* ^+^, *cmr2* ^+^, *mug113* ^+^, *usb1* ^+^, *SPAC9G1.07* ^+^, *SPBC16C6.04* ^+^, *yta6* ^+^, *SPBC660.17c* ^+^, *SPCC1494.08c* ^+^, and *SPCC14G10.04* ^+^, and all of these need to be further characterized.

**Table 1:** **Summary of osmo-remediable and osmo-irremediable phenotype of micafungin-sensitive mutants.**

Phenotype	Mutants
*Osmo-remediable micafungin sensitivity*	Δ*pmk1*, Δ*mde10*, Δ*rgf1*, Δ*rga8*, Δ*rho2*, Δ*pek1*, Δ*end4*, Δ*pvg5*, Δ*pck1*, Δ*ent3*, Δ*pab1*, Δ*yam8*, Δ*myo1*, Δ*ogm1*, Δ*rga7*, Δ*rhp54*, Δ*ccr4*, Δ*caf1*, Δ*png1*, Δ*pms1*, Δ*pbp1*, Δ*pmc6*, Δ*tup12*, Δ*ccq1*, Δ*cdt2*, Δ*trt1*, Δ*prw1*, Δ*SPCC1450.03*, Δ*mlo3*, Δ*nhp10*, Δ*mug183*, Δ*vps1302*, Δ*emc1*, Δ*imt3*, Δ*psh3*, Δ*apm1*, Δ*gyp1*, Δ*age1*, Δ*fsv1*, Δ*ecm6*, Δ*rrd2*, Δ*rrd1*, Δ*tco89*, Δ*ste20*, Δ*plc1*, Δ*rnc1*, Δ*vac7*, Δ*pub1*, Δ*SPAC328.02*, Δ*ubr1*, Δ*ubi1*, Δ*cyb5*, Δ*are2*, Δ*ckb1*, Δ*ddb1*, Δ*oca3*, Δ*mug123*, Δ*cip2*, Δ*aap1*, Δ*SPBC1861.05*, Δ*met3*, Δ*dad5*, Δ*rtc3*, Δ*put2*, Δ*SPBC4F6.11c*,Δ*mug113*, Δ*usb1*, Δ*SPAC9G1.07*, Δ*SPBC16C6.04*, Δ*SPBC660.17c*,Δ*SPCC1494.08c*, Δ*SPCC14G10.04*, Δ*vps1*, Δ*mal3*, Δ*SPBC1289.14*, Δ*tea2*, Δ*raf2*, Δ*hrp3*, Δ*trm112*, Δ*rpl3202*, Δ*mhf1*, Δ*yox1*, Δ*arp42*, Δ*yar1*, Δ*hcr1*, Δ*rpl2001*, Δ*dre4*, Δ*rpl4301*, Δ*cbp1*, Δ*rpl1702*, Δ*vps26*, Δ*nup124*, Δ*gga1*, Δ*trs85*, Δ*vam7*, Δ*npp106*, Δ*wis2*, Δ*whi2*, Δ*pnk1*, Δ*ppk5*, Δ*tor1*, Δ*SPBC32F12.07c*, Δ*mub1*, Δ*rps31*, Δ*alp14*, Δ*aim45*, Δ*mug42*, Δ*mae2*, Δ*irc6*, Δ*pdp1*, Δ*mug132*, Δ*dad1*, Δ*clp1*, Δ*SPAC13C5.04*, Δ*sol1*, Δ*pct1*, Δ*met11*, Δ*SPBC651.04*, Δ*ldh1*, Δ*SPCC1020.07*, Δ*ilm1*, Δ*pwp1*
*Osmo-irremediable micafungin sensitivity*	Δ*SPCC1322.03*, Δ*pvg2*, Δ*rhp6*, Δ*exo2*, Δ*tyw1*, Δ*pat1*, Δ*rtr1*, Δ*mms1*, Δ*yta7*, Δ*rhp51*, Δ*fbh1*, Δ*SPCC825.01*, Δ*rrp7*, Δ*rpl15*, Δ*ada2*, Δ*vps45*, Δ*tlg2*, Δ*pal1*, Δ*ivn1*, Δ*ryh1*, Δ*sst2*, Δ*dnf2*, Δ*erd1*, Δ*caf5*, Δ*SPBC1683.03c*, Δ*tdh1*, Δ*sts1*, Δ*scs7*, Δ*yps1*, Δ*SPAC22F8.04*, Δ*mug14*, Δ*mug86*, Δ*tim21*, Δ*mto1*, Δ*svf1*, Δ*cmr2*, Δ*yta6*

### Cell wall digestion assay of micafungin-sensitive mutants

**Figure 2:**
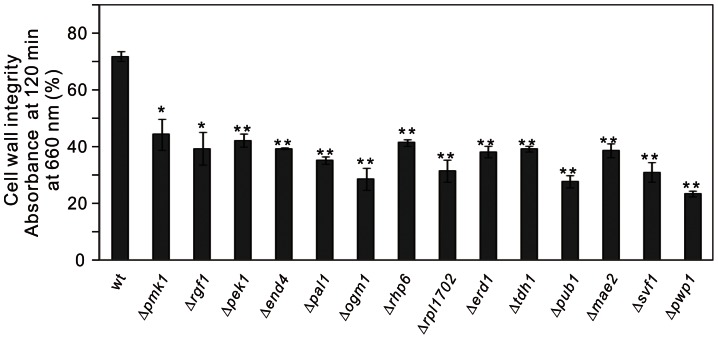
Cell wall digestion of wild-type cells and micafungin-sensitive mutants by *β*-glucanase. Cells exponentially growing in YES medium were harvested, incubated with β-glucanase at 27°C, and subjected to vigorous shaking. Cell lysis was monitored by measurements of the OD at 660 nm. The data shown are representative of triplicate experiments. The bars represent the means ± SD. Strains with statistical differences from the wild-type was marked with * on the graph, *P<0.05; **P<0.01, t-test, n = 3.

To further confirm the cell integrity defect associated with micafungin-sensitive mutants, cell wall digestion assays were performed using *β*-glucanase, another cell wall-damaging agent. Log-phase wild-type cells and micafungin-sensitive mutants were treated as described in Materials and Methods. As shown in Figure 2, the OD of wild-type cells at 120 min was decreased to 79.8%, and that of Δ*pmk1* cells was decreased to 49.2% (the value before the addition of the enzyme was taken as 100%). Our results showed that 21.3% (34/159 mutants), were lysed significantly faster than wild-type cells (with an OD lower than 60% at 120 min) (Table 2). Specifically, 13 mutants, namely, Δ*pek1*, Δ*rgf1*, Δ*pal1*, Δ*end4*, Δ*rhp6*, Δ*pub1*, Δ*svf1*, Δ*ogm1*, Δ*erd1*, Δ*rpl1702*, Δ*tdh1*, Δ*mae2*, and Δ*pwp1*, were lysed even faster than the Δ*pmk1* cells (Figure 2). Among these mutants, some showed severe sensitivity to micafungin, including Δ*pek1*, Δ*rgf1*, Δ*pal1*, Δ*end4*, Δ*rhp6*, Δ*pub1*, and Δ*svf1*. The *pek1* ^+^, *rgf1* ^+^, *pal1* ^+^, *end4* ^+^, and *rhp6* ^+^ genes have been introduced above. The *pub1* ^+^ gene is homologous to the budding yeast E3 ubiquitin ligase *RSP5*. Rsp5 affects the isoprenoid pathway which has important roles in ergosterol biosynthesis, protein glycosylation and transport and in this way may influence the composition of the plasma membrane and cell wall [34], [35]. The *svf1* ^+^ gene is a homolog of budding yeast survival factor *SVF1*, which regulates the generation of a specific subset of phytosphingosine. Cells lacking *SVF1* are hypersensitive to cold stress, menadione, acetic acid, H_2_O_2_ and other reactive oxidative species [36], [37]. Also, some mutants only showed moderate or mild sensitivity to micafungin, including Δ*ogm1*, Δ*erd1*, Δ*rpl1702*, Δ*tdh1*, Δ*mae2*, and Δ*pwp1*. Ogm1 is one of three O-glycoside mannosyltransferases and initiates the O-mannosylation in fission yeast. O-mannosylation is indispensable for cell wall integrity and normal cellular morphogenesis [38], [39]. The *erd1* ^+^ gene, is homologous to the budding yeast *ERD1*, which is required for the retention of luminal endoplasmic reticulum proteins, affects glycoprotein processing in the Golgi apparatus [40]. The *rpl1702* ^+^ gene, encoding a 60S ribosomal protein L17, is homologous to the budding yeast *RPL17A*. It has been demonstrated that the product of RPL17A increases in response to DNA replication stress [41]. Tdh1 is a GAPDH enzyme that catalyzes the sixth step of the glycolytic pathway and associates with the stress-response MAPKKKs in fission yeast [42]. The malic enzyme Mae2 assists in maintaining the intracellular redox balance and the expression of Mae2 is regulated in response to the carbon source, lack of oxygen and osmotic stress conditions [43]. Pwp1 is a cell wall protein and contains a glycosylphosphatidylinositol-anchored (GPI-anchored) domain, but its role has not been defined. These results proved again that causative genes of these mutants play important roles in maintaining cell wall integrity.

**Table 2:** **Summary of cell wall digestion assay of micafungin-sensitive mutants.**

Phenotype	Mutants
cell wall digestion assay	Δ*rgf1*, Δ*pek1*, Δ*end4*, Δ*pvg5*, Δ*pal1*, Δ*yam8*, Δ*pmk1*, Δ*pat1*, Δ*rhp6*, Δ*ccr4*, Δ*tyw1*, Δ*exo2*, Δ*caf1*, Δ*png1*, Δ*rtr1*, Δ*SPCC1450.03*, Δ*rpl3202*, Δ*rpl1702*, Δ*ada2*, Δ*tlg2*, Δ*imt3*, Δ*ogm1*, Δ*erd1*, Δ*vps45*, Δ*apm1*, Δ*tdh1*, Δ*ubi1*, Δ*pub1*, Δ*mug86*, Δ*rtc3*, Δ*mae2*, Δ*svf1*, Δ*SPBC16C6.04*, Δ*pwp1*

### Osmo-remedial phenotype of micafungin-sensitive mutants

**Figure 3:**
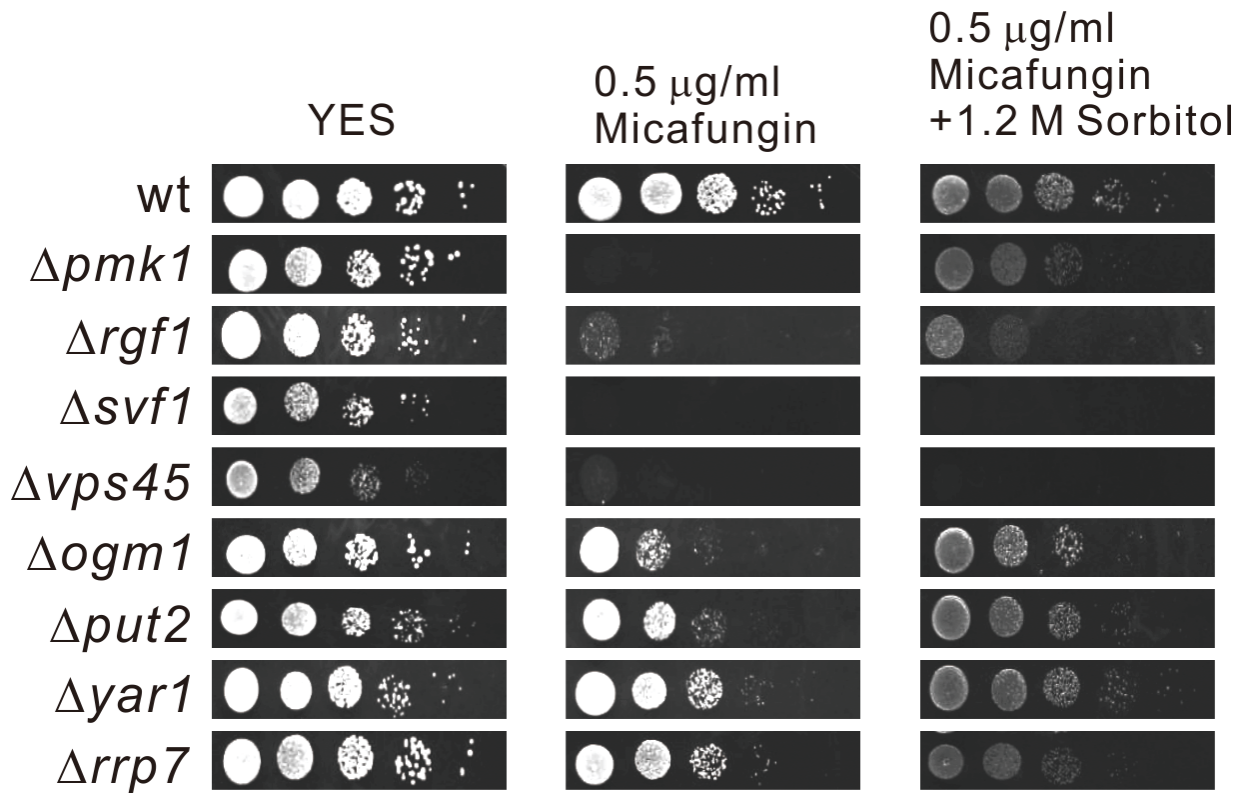
Osmo-remedial phenotype of micafungin-sensitive mutants. Wild-type cells and micafungin-sensitive mutants grown at log phase were spotted onto each plate as indicated and then incubated at 27°C for 4 days.

It has been demonstrated that defects in cell wall integrity can be compensated for by increase in the osmolarity of the growth media [44]. Here, we investigated the growth of 159 micafungin-sensitive mutants on YES plates containing both 0.5 µg/ml micafungin and 1.2 M sorbitol for the osmo-remedial phenotype (Figure 3) and found micafungin sensitivity of 122 mutants was suppressed by the presence of sorbitol (Table 1), suggesting that the increased intracellular glycerol could suppress the micafungin sensitivity induced by deletion of most of genes involved in cell wall integrity. Among the remaining 37 mutants (Table 1), some mutants showed sensitivities to sorbitol alone (data not shown), such as Δ*rhp6* and Δ*exo2*. The reasons why the other mutants were not remediated by sorbitol are not clear. Probably micafungin sensitivity of these mutants is caused not by the change of cellular turgor pressure but by the absence of localization of some important cell wall components.

### Response to other antifungal drugs of micafungin-sensitive mutants

**Figure 4:**
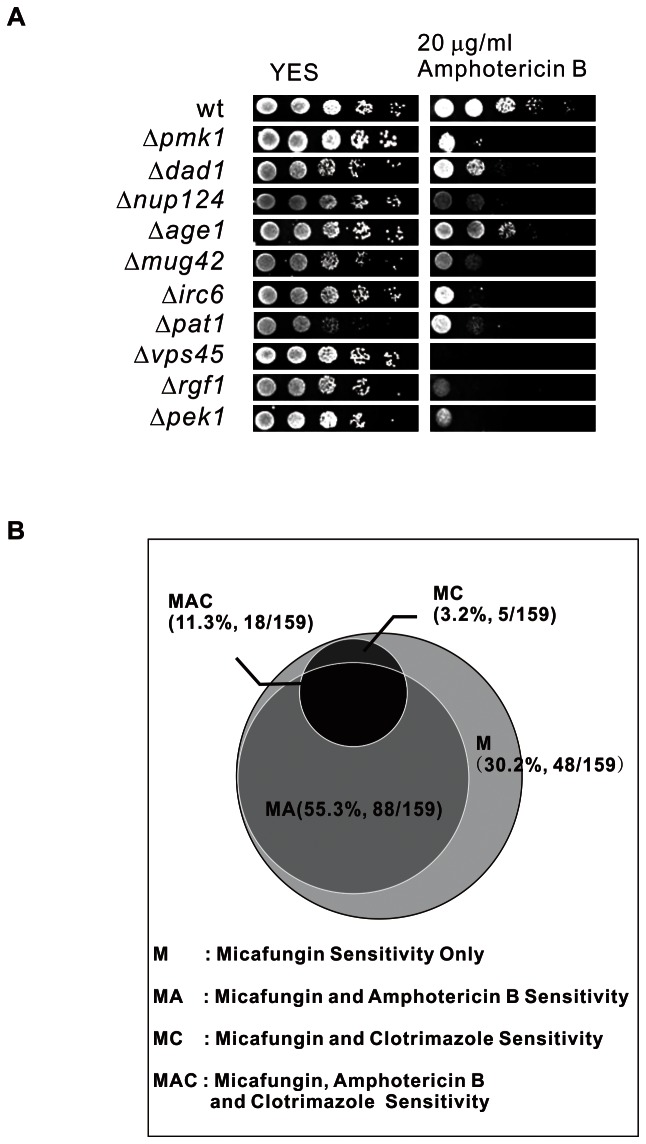
Response to other antifungal drugs of micafungin-sensitive mutants. (A) Representative examples of micafungin-sensitive mutants that showed varying levels of sensitivity to AmB. Cells were spotted onto plates containing YES or YES plus 20 µg/ml AmB and incubated at 27°C for 4 days. (B) Summary of clotrimazole-sensitive and AmB-sensitive mutants among isolated micafungin-sensitive mutants in this screening.

In our previous study, among 109 terbinafine- and clotrimazole-sensitive mutants, 34 mutants also showed hypersensitivity to polyene antifungal drug AmB [7]. Here, the growth of 159 micafungin-sensitive mutants on YES plates containing 20 µg/ml AmB were investigated (Figure 4A and Table S1).

In the present study, 14.5% of 159 micafungin-sensitive mutants (23 mutants, MC plus MAC in Figure 4B) showed clotrimazole sensitivity. And except Δ*irc6*, 22 of these 23 mutants also showed terbinafine sensitivity, indicating that these 23 causative genes are involved in ergosterol biosynthesis. A greater percentage of micafungin-sensitive mutants (66.7%, 106 mutants, MA plus MAC in Figure 4B) showed varying levels of sensitivity to AmB (Figure 4A). Our results suggested that these well-known antifungal drugs including clotrimazole, terbinafine and AmB might increase growth inhibitory activity of micafungin against their corresponding cells.

### Synergistic effects of micafungin and AmB on the growth inhibition in wild-type cells

**Figure 5:**
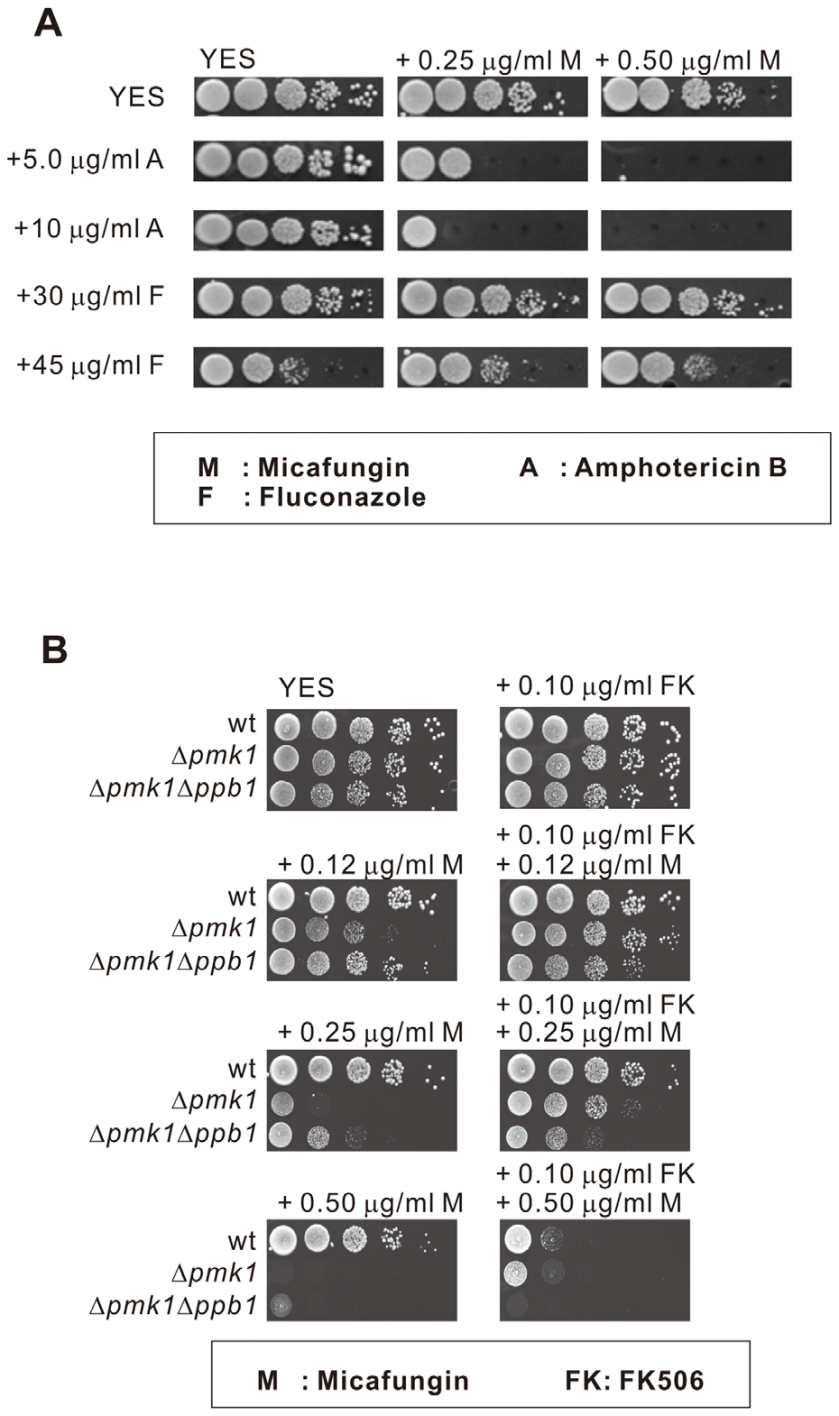
Combined inhibitory effects of micafungin on cell growth with other drugs. (A) Micafungin exhibited extremely effective inhibitory activity in combination with AmB. Wild-type cells were spotted onto each plate as indicated and incubated at 27°C for 4 days.(B) FK506 increased micafungin sensitivity of wild-type cells, whereas it attenuated the micafungin sensitivity of Δ*pmk1* cells. Cells were spotted onto each plate as indicated and incubated at 27°C for 4 days.

We investigated the interactions of micafungin with AmB and fluconazole, two major antifungal drugs used in clinical practice. We compared the growth of wild-type cells on YES plates containing micafungin alone, micafungin plus AmB, or micafungin plus fluconazole. The inhibitory activity of micafungin was obviously increased when combined with AmB, whereas there was almost no change between the growth on the plates containing micafungin alone and micafungin plus fluconazole (Figure 5A). The combination effects of micafungin plus clotrimazole, or micafungin plus terbinafine are similar with those of micafungin plus fluconazole (Data not shown). Our results demonstrated that the combination of micafungin and AmB had synergistic effects against wild-type cells, which provides useful information for the treatment of fungal infections.

### FK506 increased the growth inhibitory activity of micafungin against wild-type cells

The immunosuppressive drug FK506 has been widely used in the management of autoimmune diseases and prevention of transplant rejection, and it is usually administered in combination with antifungal drug in clinical practice [45]. Here, the growth of wild-type cells on YES plates containing both micafungin and FK506 were investigated. Results showed that FK506 increased the growth inhibitory activity of micafungin against wild-type cells (Figure 5B), indicating that FK506 has synergistic inhibitory effects with micafungin on the growth of wild-type cells. These are consistent with the results in *Aspergillus fumigatus* [46].

### FK506 decreased the growth inhibitory activity of micafungin against Δ*pmk1* cells

FK506 is a calcineurin inhibitor. In fission yeast calcineurin antagonistically acts with the Pmk1 MAPK in the regulation of cytoplasmic Ca^2+^ influx [19]. The *ppb1* ^+^ gene encodes a single catalytic subunit of fission yeast calcineurin [47]. Here, we also investigated the growth of Δ*pmk1* and Δ*pmk1*Δ*ppb1* cells on YES plates containing micafungin, FK506, and both of these two agents, respectively. Surprisingly, we found that Δ*pmk1*Δ*ppb1* cells showed a lower sensitivity to micafungin than Δ*pmk1* cells. Furthermore, contrary to the results found in wild-type cells, Δ*pmk1* cells showed an attenuated micafungin sensitivity in the presence of FK506 (Figure 5B). These results demonstrated that deletion or inhibition of calcineurin antagonized the growth-inhibitory activity of micafungin against Δ*pmk1* cells. In contrast to Δ*pmk1* cells, no growth difference of Δ*pmk1*Δ*ppb1* cells was found on the plates containing micafungin alone or containing both micafungin and FK506, suggesting FK506 exerts its function in a calcineurin-dependent manner.

In conclusion, we identified 159 mutants displaying hypersensitivity to micafungin and classified them into various functional categories. Information of the causative genes would contribute to the emerging topic of personalized medicine. On the other hand, combined applications of micafungin with some common drugs used in clinical practice were also investigated. AmB increased inhibitory activity of micafungin against wild-type cells, whereas fluconazole, clotrimazole and terbinafine did not. It is particularly interesting to note that FK506 has synergistic inhibitory effects with micafungin on the growth of wild-type cells, whereas it suppresses the inhibitory effect of micafungin against Δ*pmk1* cells. These findings provide valuable information for new potential drug combinations in the treatment of fungal infections.

## Supporting Information

Table S1 Summary of the gene name and products of micafungin-sensitive mutants.
(DOCX)

Table S2 *Schizosaccharomyces pombe* haploid strains used in this study.
(DOCX)
