# Supplementary material for: A Genome-Wide Screening of Potential Target Genes to Enhance the Antifungal Activity of Micafungin in Schizosaccharomyces pombe
Source: PLoS One. 2013 May 30;8(5):e65904. doi: 10.1371/journal.pone.0065904 (PMC3667807; doi:10.1371/journal.pone.0065904)
Supplement: Table S1 — Summary of the gene name and products of micafungin-sensitive mutants. (DOCX) [file pone.0065904.s001.docx]

**Table S1. List of micafungin-sensitive mutants**

| Micafungin | AmB | Systematic Name | Common name | Gene Description^a^ |
| --- | --- | --- | --- | --- |
| **Cell Wall Biosynthesis (15 genes)** | | | | |
| +++ | +++ | SPBC119.08 | pmk1 | MAP kinase Pmk1 |
| +++ | +++ | SPBC543.07 | pek1 | MAP kinase kinase Pek1 |
| +++ | +++ | SPCC645.07 | rgf1 | RhoGEF for Rho1, Rgf1 |
| +++ | +++ | SPAC16.01 | rho2 | Rho family GTPase Rho2 |
| +++ | ++ | SPAC13A11.01c | rga8 | GTPase activating protein Rga8 |
| +++ | - | SPAC17G8.14c | pck1 | protein kinase C (PKC)-like Pck1 |
| +++ | - | SPAC17A5.04c | mde10 | spore wall assembly peptidase Mde10 |
| +++ | - | SPAC22F8.02c | pvg5 | PvGal biosynthesis protein Pvg5 |
| +++ | ++ | SPCC1322.03 | trp1322 | membrane transporter |
| ++ | - | SPBC23G7.08c | rga7 | GTPase activating protein Rga7 |
| ++ | ++ | SPAC27E2.07 | pvg2 | galactose residue biosynthesis protein Pvg2 |
| ++ | + | SPAC227.07c | pab1 | protein phosphatase regulatory subunit Pab1 |
| ++ | - | SPAC1F5.08c | yam8 | calcium transport protein |
| ++ | - | SPBC146.13c | myo1 | myosin type I |
| ++ | + | SPAC22A12.07c | ogm1 | protein O-mannosyltransferase Ogm1 |
| **Gene Expression and Chromatin Remodeling (43 genes)** | | | | |
| +++ | +++ | SPAC15A10.03c | rhp54 | Rad54 homolog Rhp54 |
| +++ | +++ | SPAC18B11.07c | rhp6 | Rad6 homolog Rhp6 |
| +++ | + | SPCC31H12.08c | ccr4 | CCR4-Not complex subunit Ccr4 |
| +++ | +++ | SPCC18.06c | caf1 | CCR4-Not complex subunit Caf1 |
| +++ | - | SPAC17A5.14 | exo2 | exonuclease II Exo2 |
| +++ | ++ | SPAC3G9.08 | png1 | ING family homolog Png1 |
| +++ | + | SPAC19G12.02c | pms1 | MutL family mismatch-repair protein Pms1 |
| +++ | ++ | SPCC1020.08 | tyw1^c^ | wybutosine biosynthesis protein Tyw1 |
| +++ | ++ | SPBC19G7.10c | pat1^c^ | topoisomerase associated protein |
| ++ | +++ | SPAC23A1.16c | rtr1 | DUF408 family protein |
| ++ | +++ | SPBC21B10.03c | pbp1^c^ | ataxin-2 homolog |
| ++ | - | SPAC5D6.05 | pmc6 | mediator complex subunit Pmc6 |
| ++ | ++ | SPAC630.14c | tup12 | transcriptional corepressor Tup12 |
| ++ | ++ | SPCC188.07 | ccq1 | telomere maintenance protein |
| ++ | +++ | SPAC17H9.19c | cdt2 | WD repeat protein Cdt2 |
| ++ | ++ | SPAC3H8.05c | mms1 | conserved fungal protein |
| ++ | + | SPBC29A3.14c | trt1 | telomerase reverse transcriptase 1 protein Trt1 |
| ++ | ++ | SPAC29A4.18 | prw1 | Clr6 histone deacetylase complex subunit Prw1 |
| ++ | + | SPCC1450.03 | N/A | ribonucleoprotein (RNP) complex |
| ++ | - | SPBC1D7.04 | mlo3 | RNA annealing factor Mlo3 |
| ++ | + | SPCC16C4.20c | nhp10^c^ | sequence orphan |
| ++ | - | SPAC31G5.19 | yta7^c^ | ATPase with bromodomain protein |
| + | + | SPCC970.07c | raf2 | Rik1-associated factor Raf2 |
| + | - | SPAC23D3.09 | arp42 | SWI/SNF and RSC complex subunit Arp42 |
| + | - | SPBC21B10.13c | yox1 | transcription factor |
| + | ++ | SPAC644.14c | rhp51 | recombinase Rhp51 |
| + | + | SPAC3A12.13c | hcr1^c^ | translation initiation factor eIF3 complex subunit |
| + | ++ | SPBC1105.04c | cbp1 | CENP-B homolog |
| + | - | SPAC3G6.01 | hrp3 | ATP-dependent DNA helicase Hrp3 |
| + | ++ | SPBC336.01 | fbh1 | DNA helicase I |
| + | + | SPAC13C5.02 | dre4 | DNA replication protein Dre4 |
| + | ++ | SPAC31A2.02 | trm112 | tRNA (guanine-N2-)-methyltransferase regulatory subunit Trm112 |
| + | ++ | SPCC825.01 | N/A | ribosome biogenesis ATPase, Arb family |
| + | - | SPBC776.17 | rrp7^c^ | rRNA processing protein Rrp7 |
| + | - | SPAC3H5.10 | rpl3202 | 60S ribosomal protein L32 |
| + | ++ | SPAC3A12.10 | rpl2001 | 60S ribosomal protein L20a |
| + | ++ | SPBC800.04c | rpl4301 | 60S ribosomal protein L37a |
| + | + | SPCC576.11 | rpl15 | 60S ribosomal protein L15 |
| + | ++ | SPCC364.03 | rpl1702 | 60S ribosomal protein L17 |
| + | + | SPBP16F5.05c | yar1^b^ | ribosome biogenesis protein Nop8 |
| + | + | SPAC6G9.03c | mug183 | histone chaperone Rtt106-like |
| + | - | SPCC24B10.08c | ada2 | histone  acetyltransferase complex subunit Ada2 |
| + | + | SPBC2D10.16 | mhf1 | sequence orphan |
| Membrane Trafficking (28 genes) | | | | |
| +++ | + | SPBC16C6.02c | vps1302 | chorein homolog |
| +++ | +++ | SPAC2G11.03c | vps45 | vacuolar sorting protein Vps 45 |
| +++ | ++ | SPAC823.05c | tlg2 | SNARE Tlg2 |
| +++ | - | SPCC794.11c | ent3^c^ | ENTH domain protein Ent3 |
| +++ | ++ | SPAC17G8.11c | imt3 | mannosyltransferase complex subunit |
| +++ | +++ | SPCP1E11.04c | pal1 | membrane associated protein Pal1 |
| +++ | ++ | SPAC688.11 | end4 | Huntingtin-interacting protein homolog |
| +++ | + | SPAC25H1.07 | emc1 | DUF1620 family protein |
| ++ | - | SPBC409.20c | psh3 | ER chaperone SHR3 homologue Psh3 |
| ++ | - | SPBC11B10.07c | ivn1 | CDC50 domain protein |
| ++ | +++ | SPBP16F5.07 | apm1 | AP-1 adaptor complex subunit Apm1 |
| ++ | ++ | SPAC4C5.02c | ryh1 | GTPase Ryh1 |
| ++ | +++ | SPBC530.01 | gyp1 | GTPase activating protein Gyp1 |
| ++ | + | SPAC824.09c | age1^c^ | GTPase activating protein |
| ++ | ++ | SPAC19B12.10 | sst2 | human amsh protein homolog |
| ++ | + | SPAC24B11.12c | dnf2^c^ | P-type ATPase |
| ++ | + | SPAC6F12.03c | fsv1 | SNARE Fsv1 |
| ++ | - | SPCC1020.11c | ecm6 | DUF786 family protein |
| ++ | + | SPAC227.01c | erd1 | Erd1 homolog |
| ++ | + | SPBC609.04 | caf5 | spermine family transporter |
| + | - | SPAC767.01c | vps1 | dynamin family protein Vps1 |
| + | ++ | SPAC4G9.13c | vps26 | retromer complex subunit Vps26 |
| + | ++ | SPAC30D11.04c | nup124 | nucleoporin Nup124 |
| + | +++ | SPBC25H2.16c | gga1^c^ | adaptin |
| + | + | SPBC1734.07c | trs85^c^ | TRAPP complex subunit Trs85 |
| + | - | SPCC594.06c | vam7^c^ | SNARE Vam7 |
| + | + | SPCC1739.14 | npp106 | nucleoporin Npp106 |
| + | ++ | SPBC1683.03c | N/A | membrane transporter |
| Signaling Transduction (13 genes) | | | | |
| +++ | + | SPAC4F10.04 | rrd1 | protein phosphatase type 2A, intrinsic regulator |
| +++ | ++ | SPAC1782.05 | rrd2^c^ | phosphotyrosyl phosphatase activator homolog |
| +++ | - | SPCC162.12 | tco89 | sequence orphan |
| ++ | +++ | SPBC12C2.02c | ste20 | sterility protein Ste20 |
| ++ | - | SPAC22F8.11 | plc1 | phosphoinositide phospholipase C Plc1 |
| ++ | - | SPCC757.09c | rnc1 | RNA-binding protein that suppresses calcineurin deletion Rnc1 |
| ++ | - | SPBP8B7.13 | vac7 | conserved fungal protein |
| + | + | SPBC32F12.11 | tdh1 | glyceraldehyde-3-phosphate dehydrogenase Tdh1 |
| + | - | SPAC1B3.03c | wis2 | cyclophilin family peptidyl-prolyl cis-trans isomerase Wis2 |
| + | ++ | SPBP4H10.16c | whi2^c^ | phosphatase activator |
| + | + | SPAC23C11.04c | pnk1 | DNA kinase/phosphatase Pnk1 |
| + | + | SPAC16C9.07 | ppk5 | serine/threonine protein kinase Ppk5 |
| + | + | SPBC30D10.10c | tor1 | phosphatidylinositol kinase Tor1 |
| Ubiquitination (7 genes) | | | | |
| +++ | ++ | SPAC11G7.02 | pub1 | ubiquitin-protein ligase E3 |
| ++ | +++ | SPAC328.02 | N/A | Ariadne homolog |
| ++ | + | SPBC19C7.02 | ubr1 | N-end-recognizing protein Ubr1 |
| ++ | - | SPAC11G7.04 | ubi1 | ribosomal-ubiquitin fusion protein Ubi1 |
| + | - | SPBC32F12.07c | N/A | ubiquitin-protein ligase E3 |
| + | - | SPBC31F10.10c | mub1^c^ | zf-MYND type zinc finger protein |
| + | + | SPAC589.10c | rps31^c^ | ribomal-ubiquitin fusion protein Ubi5 |
| Ergosterol Biosynthetic Process (3 genes) | | | | |
| +++ | - | SPBC29A10.16c | cyb5^c^ | cytochrome b5 |
| +++ | + | SPAC20G4.07c | sts1 | C-24(28) sterol reductase Sts1 |
| ++ | ++ | SPCP1E11.05c | are2 | sterol O-acyltransferase |
| Variety of Other Known Functions (34 genes) | | | | |
| +++ | + | SPAC19G12.08 | scs7 | fatty acid hydroxylase |
| +++ | ++ | SPAC1851.03 | ckb1 | CK2 family regulatory subunit |
| +++ | ++ | SPAC17H9.10c | ddb1 | damaged DNA binding protein Ddb1 |
| +++ | + | SPCC1795.09 | yps1 | aspartic protease Yps1 |
| ++ | + | SPBC15C4.01c | oca3 | TPR repeat protein Oca3 |
| ++ | + | SPCC16C4.17 | mug123 | meiotically upregulated gene Mug123 |
| ++ | +++ | SPAC12G12.03 | cip2 | RNA-binding protein Cip2 |
| ++ | - | SPAC4F10.02 | aap1 | aspartyl aminopeptidase |
| ++ | - | SPBC1861.05 | N/A | carbohydrate kinase |
| ++ | - | SPBC15D4.09c | met3 | cystathionine gamma-synthase |
| ++ | - | SPAC22F8.04 | N/A | triose phosphate transporter |
| ++ | + | SPBC359.06 | mug14 | adducin |
| ++ | - | SPAC5D6.09c | mug86 | acetate transporter |
| ++ | + | SPCC417.02 | dad5 | DASH complex subunit Dad5 |
| ++ | + | SPBC21C3.19 | rtc3^c^ | DUF1960 family protein |
| ++ | - | SPBC24C6.04 | put2^c^ | delta-1-pyrroline-5-carboxylate dehydrogenase |
| ++ | - | SPBC1289.09 | tim21 | mitochondrial inner membrane presequence translocase complex subunit Tim21 |
| ++ | - | SPCC417.07c | mto1 | MT organizer Mto1 |
| ++ | - | SPBC4F6.11c | N/A | asparagine synthase |
| + | + | SPCC895.07 | alp14 | Mad2-dependent spindle checkpoint component |
| + | ++ | SPBC1604.20c | tea2 | kinesin-like protein Tea2 |
| + | + | SPAC18G6.15 | mal3 | EB1 family Mal3 |
| + | + | SPAC27D7.06 | aim45^c^ | electron transfer flavoprotein alpha subunit |
| + | - | SPAC11G7.06c | mug132 | S. pombe specific UPF0300 family protein 3 |
| + | ++ | SPCC584.12 | mug42 | sequence orphan |
| + | - | SPCC794.12c | mae2 | malic enzyme |
| + | +++ | SPAC19A8.11c | irc6^c^ | recombination protein Irc6 |
| + | - | SPBC29A3.13 | pdp1 | PWWP domain protein |
| + | + | SPAC16A10.05c | dad1 | DASH complex subunit Dad1 |
| + | + | SPAC1782.09c | clp1 | Cdc14-related protein phosphatase Clp1/Flp1 |
| + | + | SPAC13C5.04 | N/A | glutamine amidotransferase |
| + | - | SPCC16C4.10 | sol1^c^ | 6-phosphogluconolactonase |
| + | - | SPCC1827.02c | pct1^c^ | cholinephosphate cytidylyltransferase |
| + | - | SPAC343.10 | met11 | methylenetetrahydrofolate reductase Met11 |
| Unknown Functions (16 genes) | | | | |
| +++ | +++ | SPCC584.11c | svf1^b^ | Svf1 family protein Svf1 |
| +++ | - | SPAC56F8.02 | cmr2^c^ | AMP binding enzyme |
| +++ | + | SPAC3F10.05c | mug113 | DUF1766 family protein |
| ++ | +++ | SPAC23C11.10 | usb1 | conserved eukaryotic protein |
| ++ | - | SPAC9G1.07 | N/A | sequence orphan |
| ++ | ++ | SPBC16C6.04 | N/A | sequence orphan |
| ++ | - | SPAC328.04 | yta6^c^ | AAA family ATPase, unknown biological role |
| ++ | + | SPBC660.17c | N/A | sequence orphan |
| ++ | ++ | SPCC1494.08c | N/A | conserved fungal protein |
| ++ | + | SPCC14G10.04 | N/A | sequence orphan |
| + | - | SPBC1289.14 | N/A | adducin |
| + | + | SPBC651.04 | N/A | sequence orphan |
| + | - | SPAC57A10.08c | ldh1^c^ | esterase/lipase |
| + | - | SPCC1020.07 | N/A | haloacid dehalogenase-like hydrolase |
| + | ++ | SPAC1782.02c | ilm1^c^ | conserved fungal protein |
| + | - | SPCC1322.10 | pwp1 | conserved fungal protein |

NOTE:

+++, severely sensitive; ++moderately sensitive; +, mildly sensitive; -, not sensitive.

^c^ means that the common name is taken from the orthology of *S. cerevisae*

N/A indicates that common gene name is not applicable.
