# Supplementary material for: A Genome-Wide Screening of Potential Target Genes to Enhance the Antifungal Activity of Micafungin in Schizosaccharomyces pombe
Source: PLoS One. 2013 May 30;8(5):e65904. doi: 10.1371/journal.pone.0065904 (PMC3667807; doi:10.1371/journal.pone.0065904)
Supplement: Table S2 — Schizosaccharomyces pombe haploid strains used in this study. (DOCX) [file pone.0065904.s002.docx]

**Table S2. *Schizosaccharomyces pombe* haploid strains used in this study.**

| **Strain** | **Genotype** |
| --- | --- |
| KP251 | *h^-^ leu1-32 ura4-D18 pmk1*::*ura4^+^ ppb1*::*ura4^+^* |
